# Decolonizing higher education staffing in the UK: addressing racial and gendered inequalities through deliberate EDI

**DOI:** 10.3389/fsoc.2025.1693275

**Published:** 2025-12-03

**Authors:** Obasanjo Bolarinwa

**Affiliations:** 1 Department of Global Healthcare Management, York St John University, London, United Kingdom; 2 Demography and Population Studies Programme, Schools of Public Health and Social Sciences, University of the Witwatersrand, Johannesburg, South Africa

**Keywords:** higher education, EDI, United Kingdom, racial, gender

## Introduction

Decolonization in higher education has gained significant attention in the UK, particularly in response to calls for greater inclusivity, diversity, and equity in academic institutions. While much focus has been placed on decolonising curricula, there is a growing recognition that decolonizing the staff body is equally critical toward addressing systemic disparities and dismantling colonial legacies entrenched within academic institutions, thereby promoting truly equitable teaching and learning environments. This commentary discusses how deliberate Equality, Diversity, and Inclusion (EDI) strategies can contribute to addressing the historical and systemic imbalances in staffing within the UK's higher education institutions, considering both racial and gender disparities.

## The case for decolonizing higher education staff

Higher education institutions in the UK remain largely shaped by Eurocentrism and historical legacies that have privileged certain groups while marginalizing others. Historically, contributions from minority groups and underrepresented communities, especially migrant women from the Global South, in the UK's higher educational system are often overlooked (Belluigi et al., 2024), contributing to persistent marginalization and lack of diversity. Although student diversity has generally increased over the years, staff representation remains disproportionately White, particularly in senior academic and leadership positions (Higher Education Statistics Agency, 2025). For instance, as of the 2022/2023 academic year, Black, Asian, and Minority Ethnic (BAME) academics remain largely underrepresented in professorial roles in the UK's higher educational institutions, accounting for only 13% of professors (Higher Education Statistics Agency, 2024). Additionally, recent data revealed that Black academics still make up less than 1% of UK professors (Higher Education Statistics Agency, 2025). This highlights the continuous underrepresentation of BAME individuals in academic leadership positions despite the increasing calls to decolonize the staff body in higher educational institutions in the UK.

Although BAME representation in academic and leadership roles remains low if employed, it is highly represented in lower-paid, insecure, non-academic, and non-professional positions, highlighting the structural inequalities in the UK's higher education workforce (Higher Education Statistics Agency, 2025). Evidence shows that many universities in the UK employ a disproportionately high number of BAME individuals in cleaning, catering, and support roles, reinforcing racialized hierarchies within institutions (Osho and Alormele, 2024). This structural imbalance raises concerns about whether diversity efforts are truly addressing systemic inequalities or merely reproducing a racial division of labor (Osho and Alormele, 2024).

Moreover, when analyzing staffing from a gendered perspective, Black women and women of color face dual discrimination, both as racial minorities and as women (Crenshaw, 1989). They are less likely to be promoted to senior academic positions and are more likely to be concentrated in lower-paid, insecure contracts compared to their White and male counterparts (Osho and Alormele, 2024; Rollock, 2019). Although the narrative around the total number of women in higher education in the UK is gradually improving, with some institutions employing more women than men, the intersection of race and gender exacerbates barriers to career progression, contributing to the persistent lack of representation in higher education leadership (Rollock, 2019). For instance, females account for only 32% of professors in the UK as of the 2023/2024 academic year (Higher Education Statistics Agency, 2025). The low representation of women in academic leadership positions in the UK is particularly pronounced in Russell Group universities (Rollock, 2019), suggesting a lack of prioritization of the decolonization agenda involving racial minority staff in those institutions. Although these institutions often develop policy strategies indicating their commitment to diversity, these policies are not far-reaching and can merely be described as tokenistic (Osho and Alormele, 2024), emphasizing the need for deliberate EDI approaches in promoting equity and diversity in higher education staffing in the UK.

## The role of deliberate EDI strategies

To effectively decolonize staff composition in UK universities, institutions must go beyond symbolic diversity statements and engage in deliberate, systemic changes. These changes should address power asymmetries by promoting diversity, inclusivity, and equity in staffing, decision-making, and knowledge production beyond the tokenistic approach (Tamimi et al., 2024). Thus, decolonizing staff should not be limited to only increasing representation but also providing an inclusive environment where diverse perspectives are valued and assimilated into institutional policies and practices. Therefore, there is a need to address unconscious biases, provide equitable opportunities for career progression, and prioritize anti-racism agendas in UK universities (Tamimi et al., 2024; Tight, 2024). The following EDI strategies are essential in addressing the historical and systemic imbalances in staffing within the UK's higher education.

### Targeted recruitment and progression policies

Higher education institutions are encouraged to adopt affirmative recruitment strategies that ensure diverse representation on hiring panels and incorporate training on unconscious bias, including sexism and racism (McDuff et al., 2018). In practice, this could involve mandatory inclusion of at least one BAME academic on shortlisting and interview panels, transparent promotion criteria that account for teaching, research, and leadership contributions, and the introduction of ring-fenced leadership development programmes for underrepresented groups. Institutions could also establish annual equity audits to monitor hiring and promotion data, ensuring accountability in the implementation of these policies.

To address persistent inequalities in career advancement, institutions should also develop pipeline programmes that support the progression of BAME staff and actively tackle the barriers they face in promotion and leadership pathways. Such programmes could include structured mentorship, sponsorship by senior leaders, and targeted funding opportunities for BAME early-career researchers to build competitive portfolios for promotion. Moreover, efforts to improve diversity must extend beyond non-academic roles to meaningfully increase BAME representation in academic teaching positions as well (McDuff et al., 2018).

The urgency of implementing these measures is underscored by current policy and societal drivers. The Equality Act 2010, the growth of Advance HE's Race Equality Charter, and increased accountability demands from UKRI and other funding councils highlight a shifting landscape in which diversity and inclusion are no longer optional but central to institutional competitiveness. The post-2020 global racial justice movements and increasing student activism around representation further add momentum to the call for change.

### Mentorship and leadership development

Establishing formal mentorship schemes for early-career academics from underrepresented backgrounds can play a crucial role in addressing disparities in career progression (Rollock, 2019). Structured mentorship could involve pairing junior BAME academics with senior faculty through transparent schemes that include clear objectives, regular meetings, and progress evaluations. These programmes should be institutionally supported rather than voluntary add-ons, with recognition in workload models to ensure mentors are adequately resourced. In parallel, sponsorship models, where senior leaders actively advocate for the career advancement of mentees, can complement traditional mentorship.

Tailored leadership development programmes for BAME women in academia are also vital, as they provide the skills, confidence, and networks needed to access senior roles. These could include targeted workshops on leadership competencies, opportunities for shadowing senior executives, and access to external leadership fellowships. Embedding these initiatives within institutional promotion frameworks ensures they are not treated as peripheral but as pathways to substantive career progression.

In addition, institutions should extend mentorship opportunities to BAME staff in non-academic roles, fostering career mobility into both professional and academic pathways (Rollock, 2019). By supporting upward mobility, universities can begin to dismantle the racialized division of labor that currently relegates many BAME staff to low-paid, insecure roles.

The urgency of developing these mentorship and leadership pathways is underscored by persistent evidence of the “leaky pipeline” in UK academia, where BAME academics, especially Black women, enter the profession but are systematically filtered out before reaching senior levels. The current climate of heightened scrutiny on institutional EDI performance, coupled with student-led demands for greater representation, makes inaction increasingly untenable.

### Decolonizing institutional cultures

Higher education institutions must take a critical look at their workplace culture, policies, and governance structures to uncover and address embedded biases, with a focus on dismantling colonial legacies and hierarchies that persist within academia (Bhopal and Henderson, 2021). This process should be supported by mandatory training for staff on anti-racism, inclusivity, and decolonization, shifting from symbolic gestures to genuine institutional transformation. It is also crucial that diversity initiatives recognize and respond to the specific challenges experienced by BAME women, ensuring that gendered racism is not overlooked in policy and practice (Bhopal and Henderson, 2021).

Higher education institutions must take a critical look at their workplace culture, policies, and governance structures to uncover and address embedded biases, with a focus on dismantling colonial legacies and hierarchies that persist within academia (Bhopal and Henderson, 2021). Practical steps could include mandatory training for all staff on anti-racism, inclusivity, and decolonization; reforms to recruitment and promotion panels to ensure diverse representation; and the incorporation of equity criteria into decision-making processes at departmental and institutional levels. In addition, regular equity audits and anonymous staff surveys could provide evidence to track cultural change and highlight areas requiring targeted intervention.

This process should be supported by mandatory training for staff on anti-racism, inclusivity, and decolonization, shifting from symbolic gestures to genuine institutional transformation. The urgency of embedding such initiatives is reflected in recent societal pressure, including global movements for racial justice, heightened student activism demanding greater accountability from universities, and public scrutiny of institutional diversity performance. These developments signal that universities can no longer rely on symbolic commitments to equality but must demonstrate tangible cultural change.

It is also crucial that diversity initiatives recognize and respond to the specific challenges experienced by BAME women, ensuring that gendered racism is not overlooked in policy and practice (Bhopal and Henderson, 2021).

### Embedding EDI in performance metrics

Universities should embed EDI initiatives into performance reviews and institutional benchmarks to ensure accountability in recruitment, retention, and promotion processes. Funding bodies and accreditation agencies also have a role to play by incentivizing inclusive hiring practices and supporting research collaborations that elevate diverse voices (McDuff et al., 2018). In addition, institutions should undertake regular equity audits to monitor the representation of BAME staff across various employment categories and implement strategies to address identified disparities (Bhopal and Henderson, 2021).

Universities should embed EDI initiatives into performance reviews and institutional benchmarks to ensure accountability in recruitment, retention, and promotion processes. This could be achieved through the development of equity scorecards that monitor progress across departments, annual equity audits that track staff representation by role and level, and the integration of diversity targets into institutional funding models. Linking departmental budgets or leadership performance appraisals to measurable EDI outcomes would provide strong incentives for genuine change. Funding bodies and accreditation agencies also have a critical role to play by requiring demonstrable progress on diversity as part of grant eligibility and by supporting research collaborations that elevate underrepresented voices (McDuff et al., 2018).

In addition, institutions should undertake regular equity audits to monitor the representation of BAME staff across various employment categories and implement strategies to address identified disparities (Bhopal and Henderson, 2021). The urgency of embedding these mechanisms now reflects the growing expectations of funders, professional bodies, and students, who are increasingly holding universities accountable for their diversity performance. Recent policy shifts, such as UKRI's EDI strategy, as well as the expansion of the Race Equality Charter, have created external pressure on universities to move beyond rhetorical commitments and to deliver quantifiable outcomes. Demonstrating progress on these metrics is becoming a key determinant of institutional competitiveness and credibility.

## Challenges and considerations

While deliberate EDI policies provide a pathway toward staff decolonization, challenges persist. Targeted recruitment and promotion reforms often encounter resistance framed around notions of meritocracy, where affirmative policies are misrepresented as lowering standards. Mentorship and leadership initiatives can place a disproportionate burden on the limited pool of senior BAME academics, resulting in what has been described as the “minority tax.” Similarly, embedding EDI in institutional performance frameworks can falter if accountability structures are weak, data quality is inconsistent, or reporting systems are fragmented. In addition, institutional resistance, rigid policies, lack of leadership support or commitment, non-allocation of resources, tokenism, and the risk of diversity fatigue can undermine efforts (Rollock, 2019). To counter these challenges, universities must embed decolonization in long-term structural reforms, allocate adequate resources, and implement transparent accountability mechanisms that track progress in a meaningful way rather than as a compliance exercise.

There is also a need for intersectional approaches that consider how race, gender, disability, and socioeconomic status intersect in academic career trajectories (Crenshaw, 1989). For example, policies must address the unique challenges faced by Black women and women of color, who experience compounded barriers in promotion and leadership pathways due to gendered racism.

Additionally, more robust career progression opportunities should be provided for BAME staff in non-academic roles, allowing upward mobility into professional and academic positions (Osho and Alormele, 2024). This could involve creating structured transition pathways that enable staff in administrative or support roles to pursue further qualifications, take part in professional development schemes, or access internal fellowships that bridge them into academic tracks. Institutions could also establish targeted secondment schemes and leadership apprenticeships that prepare BAME staff for managerial and academic positions. Such initiatives are critical now in light of current labor market dynamics, government EDI policy pushes, and heightened scrutiny of occupational hierarchies within universities. Without tackling these hierarchies and offering mobility into professional and academic positions, diversity efforts risk reproducing the same inequities they seek to dismantle.

## Conclusion

Decolonizing higher education staff in the UK requires a deliberate commitment to EDI and systemic changes that go beyond rhetoric and address structural inequalities in recruitment, progression, and institutional culture, thereby promoting diversity and inclusivity in higher education. By implementing targeted policies, mentorship schemes, leadership development, and accountability measures, UK universities can create a more inclusive and representative academic workforce. True transformation will only occur when diversity in staffing is recognized not just as an ethical obligation but as a core component of academic excellence and institutional success. Without tackling the disproportionate employment of BAME individuals in non-academic roles and addressing gendered racial disparities, the decolonization of higher education staffing in the UK will remain incomplete.
